# Relationship between polymorphisms in vitamin D metabolism-related genes and the risk of rickets in Han Chinese children

**DOI:** 10.1186/1471-2350-14-101

**Published:** 2013-09-30

**Authors:** Yuling Zhang, Shufen Yang, Ye Liu, Lihong Ren

**Affiliations:** 1 Department of Pediatrics, Second Affiliated Hospital of Harbin Medical University, Harbin, Heilongjiang Province, China; 2 Department of Immunology, Harbin Medical University, Harbin, Heilongjiang Province, China

**Keywords:** *GC*, *CYP2R1*, *DHCR7/NADSYN1*, Rickets, Polymorphism

## Abstract

**Background:**

Vitamin D deficiency rickets is common in China. Genetic factors may play an important role in the susceptibility to rickets. Our study aimed to identify the relationship between three vitamin D-related genes (group specific component [*GC*], cytochrome P450, family 2, subfamily R, polypeptide 1 (*CYP2R1*), and 7-dehydrocholesterol reductase/nicotinamide-adenine dinucleotide synthetase 1 (*DHCR7/NADSYN1*) and rickets in Han Chinese children from northeastern China.

**Methods:**

A total of 506 Han children from northeastern China were enrolled in the current study. Twelve SNPs in three candidate genes were genotyped using the SNaPshot assay. Linear regression was used to examine the effect of 12 single-nucleotide polymorphisms (SNPs) on the risk of rickets.

**Results:**

In our case–control cohort, six alleles of the 12 SNPs conferred a significantly increased risk of rickets in *GC* (rs4588 C, *P* = 0.003, OR: 0.583, 95% CI: 0.412-0.836; rs222020 C, *P* = 0.009, OR: 1.526, 95% CI: 1.117-2.0985; rs2282679 A, *P* = 0.010, OR: 0.636, 95% CI: 0.449-0.900; and rs2298849 C, *P* = 0.001, OR: 1.709, 95% CI: 1.250-2.338) and in *CYP2R1* (rs10741657 G, *P* = 0.019, OR: 1.467, 95% CI: 1.070-2.011; and rs2060793 G, *P* = 0.023, OR: 0.689, 95% CI: 0.502-0.944). The results remained significant after adjustment for sex and body mass index. We further analyzed the effect of genotypes under three different genetic models. After using Bonferroni’s method for multiple corrections, rs4588, rs2282679, and rs2298849 of the *GC* gene were significantly associated with rickets under the dominant (*P* =0.003 for rs4588, *P* =0.024 for rs2282679, and *P* =0.005 for rs2298849) and additive models (*P* = 0.006 for rs4588, *P* = 0.024 for rs2282679, and *P* = 0.005 for rs2298849). Haplotype analysis showed that the CAT haplotype of the *GC* gene (*P* = 0.005) and the GAA haplotype of the *CYP2R1* gene (*P* = 0.026) were associated with susceptibility to rickets.

**Conclusions:**

This case–control study confirmed the strong effect of *GC* and *CYP2R1* loci on rickets in Han children from northeastern China.

## Background

Vitamin D deficiency rickets is related to limited exposure to sunlight, poor nutrition and/or decreased dietary intake of vitamin D. Although people are encouraged to obtain adequate exposure to sunlight and fortified milk with vitamin D supplements, this disease remains common and has the potential to produce an epidemic outbreak in children in developing countries, such as China [1,2]. The prevalence of rickets among rural Chinese children was reported to be 26.7% [3].

Recent studies have indicated that genetic factors may play an important role in the susceptibility to rickets. However, only a few studies have examined the association between the vitamin D receptor (VDR) and rickets [4-7]. The most frequently studied loci include ApaI, TaqI, BsmI, and FokI, based on small sample sizes, with inconsistent results. Giampiero et al. (2008) examined BsmI and FokI in 98 Middle Eastern rachitic subjects and found that *VDR* genotypes may predispose the population to rickets by an increasing frequency of the F allele. Kaneko et al. (2007) tested the association of ApaI, TaqI, and BsmI with the risk of rickets in 80 children aged 7–10 years in Mongolia with a history of rickets, without any possible results. Several studies [8-13] also examined the association between VDR and rickets in Chinese subjects.

The group specific component (*GC*), cytochrome P450, family 2, subfamily R, polypeptide 1 (*CYP2R1*), and 7-dehydrocholesterol reductase/nicotinamide-adenine dinucleotide synthetase 1 (*DHCR7/NADSYN1*) genes are all involved in vitamin D metabolism and/or transportation [14-16]. These three genes are associated with 25(OH)D concentrations [17-20]. The effect of the genes on the development of rickets remains unclear.

This study aimed to investigate the association between *GC*, *CYP2R1*, and *DHCR7/NADSYN1* and rickets in northeastern Han Chinese children.

## Methods

The study subjects consisted of 506 northeastern Han Chinese children. Samples were collected from Harbin, a city in northeastern China. All participants were permanent residents and without consanguinity in three generations. Children were divided into two groups. One group was recruited from the outpatient clinic of the Second Affiliated Hospital, Harbin Medical University, and subjects showed obvious clinical symptoms and these were radiologically confirmed. Diagnosis of rickets was based on at least one of the following clinical signs: craniotabes, rachitic rosary, Harrison’s groove, delayed closure of the anterior fontanelle, delayed dentition, muscular hypotonia, abdomen of a frog, spinal deformation, pigeon chest, enlarged wrists, and bowed legs.

The other group was the control cohort, which comprised of healthy children with no symptoms or signs of rickets or history of rickets. These children were all involved in a randomized citywide survey of vitamin D status. Subjects who had been treated with vitamin D (except for a prophylactic supplementation dose, 400 IU/d), calcium, or medications known to interfere with calcium-phosphate metabolism were excluded.

The study protocol was approved by the Ethics Committee of Harbin Medical University. Written informed consent was obtained from all the parents of participants.

### Measurement of vitamin D levels

An enzyme-linked immunosorbent assay was conducted according to the manufacturer’s instructions (R&D Systems, Minneapolis, MN, USA) to examine plasma 25(OH)D levels. Other possible factors related to plasma 25(OH)D levels were collected, such as age, sex, height, weight, measurement data, and habitual vitamin D supplementation (400 IU/d). Information of the participants is shown in Table 1.

**Table 1:** Clinical characteristics of the participants

**Characteristic**	**Mean ± SD**	
	**Case**	**Control**
Number	105	401
Male/female	52/53	251/150
Age (years)	1.77 ± 1.07	7.17 ± 3.89^**△**^
BMI (kg/m2)	18.43 ± 4.21	18.14 ± 3.37
Calcium (mmol/L)	2.33 ± 0.16	2.42 ± 0.13^**△**^
Phosphate (mmol/L)	1.69 ± 0.24	1.67 ± 0.19
Alkaline phosphatase (IU/L)	207.33 ± 54.64	231.72 ± 69.52^**△**^
Serum 25(OH)D (ng/ml)^a^	11.82 ± 6.95	23.58 ± 8.20^**△**^
Regular vitamin D use^b^		
Yes	19	193^**△**^
No	86	208

^a^Serum collected from November to April, 2010.

^b^Intake of 400 IU/d.

^**△**^Significant P values are indicated by a triangle (^**△**^*P* < 0.05).

### Genotyping

The loci selected for this study included *GC* (rs4588, rs7041, rs222020, rs2282679, and rs2298849), *CYP2R1* (rs10741657, rs10766197, rs12794714, rs1562902, and rs2060793), and *DHCR7/NADSYN1* (rs3829251 and rs12785878), all of which have been shown to synthesize and transport vitamin D. These factors are associated with vitamin D levels.

Genomic DNA was extracted from peripheral blood leukocytes using the QIAamp DNA Blood Kit (Qiagen). Twelve single-nucleotide polymorphisms (SNPs) were genotyped using the SNaPshot assay. Primer3 (http://frodo.wi.mit.edu/) was used to design primers to amplify a different-sized fragment for each SNP within a multiplex. Extension primers, again differing in length within a multiplex, were chosen from the sequence immediately up- or down-stream of each SNP. Primer interactions within the multiplex were evaluated and minimized using the AutoDimer program (http://www.cstl.nist.gov/biotech/strbase/AutoDimerHomepage/AutoDimerProgramHomepage.htm). Polymerase chain reaction (PCR) contained 10–50 ng of DNA, 1 × HotStarTaq buffer, 3 mM MgCl_2_, 300 μM of each dNTP, 0.08 μM of each primer, and one unit of HotStarTaq polymerase (Qiagen) in a 20-μl reaction volume. The following touchdown PCR program was used: denaturation at 95°C for 15 min, followed by 11 cycles of 94°C for 20 sec, annealing at 65°C for 40 sec (decreasing by 0.5°C per cycle), and extension at 72°C for 90 sec. This was followed by 24 cycles of denaturation at 94°C for 20 sec, annealing at 59°C for 30 sec, and extension at 72°C for 90 sec, and a final extension at 72°C for 5 min. The PCR products were purified by treatment with Exonuclease I (USB Corporation) and shrimp alkaline phosphatase (USB Corporation) at 37°C for 1 h, followed by incubation at 75°C for 15 min. The extension reaction contained 1 × ABI Prism SNaPshot Multiplex Ready Reaction Mix (Applied Biosystems), 0.5 μM of each primer, and 1 μl of each PCR product, and was carried out as recommended (Applied Biosystems). The extension PCR products were purified using 1 unit of shrimp alkaline phosphatase and then analyzed using an ABI 3130×l Genetic Analyzer. SNP calling was carried out using GeneMapperTM software v.4.0 (Applied Biosystems). For quality control, genotyping was performed without the knowledge of case/control status of the subjects, and a 5% random sample of cases and controls was genotyped twice per SNP for all SNPs by different people; the reproducibility was 100%.

### Statistical analysis

To examine the effect of the variants on plasma 25(OH)D levels, linear regression was performed after adjusting for sex and body mass index (BMI). The analyses were performed using dominant, additive, and recessive genetic models. Bonferroni’s method was applied to correct for multiple testing. Haplotype frequencies for different genes were estimated using the expectation-maximization method by Haploview 4.0 software. Statistical analyses were performed using SPSS software (version 17.0; SPSS, Chicago, IL, USA). Data were analyzed using two-sided *P* values. The Hardy-Weinberg equilibrium was evaluated using Pearson’s χ^2^ test (Table 2). Statistical power was assessed using the Genetic Power Calculator [21].

**Table 2:** Association of 12 SNPs with rickets in cases and controls

**Gene**	**SNP**	**MAF**	***p*for HWET**	**A/a**	**Allele-specific (unadjusted)**		**Allele-specific (adjusted)**	
					***p*value**	**OR (95% CI)**	***p*value**^**b**^	**OR (95% CI)**
GC	rs4588	0.31	0.30	C^a^/A	**0.003**^**△**^	0.587 (0.412-0.836)	**0.003**^**△**^	0.583 (0.409-0.833)
	rs7041	0.26	0.29	T/G^a^	0.252	1.216 (0.867-1.705)	0.167	1.272 (0.901-1.791)
	rs222020	0.34	0.67	T/C^a^	**0.009**^**△**^	1.526 (1.117-2.085)	**0.009**^**△**^	1.517 (1.108-2.078)
	rs2282679	0.32	0.22	A^a^/C	**0.010**^**△**^	0.636 (0.449-0.900)	**0.010**^**△**^	0.631 (0.444-0.895)
	rs2298849	0.32	0.98	T/C^a^	**0.001**^**△**^	1.709 (1.250-2.338)	**0.001**^**△**^	1.722 (1.256-2.362)
CYP2R1	rs10741657	0.43	0.25	G^a^/A	**0.019**^**△**^	1.467 (1.070-2.011)	**0.016**^**△**^	1.477 (1.075-2.030)
	rs10766197	0.45	0.36	G^a^/A	0.374	0.855 (0.619-1.179)	0.416	0.874 (0.632-1.209)
	rs12794714	0.43	0.36	G/A^a^	0.089	1.322 (0.968-1.804)	0.075	1.330 (0.971-1.820)
	rs1562902	0.47	0.46	T^a^/C	0.120	1.279 (0.940-1.740)	0.115	1.284 (0.941-1.751)
	rs2060793	0.27	0.42	G^a^/A	**0.023**^**△**^	0.689 (0.502-0.944)	**0.019**^**△**^	0.684 (0.498-0.940)
DHCR7/NADSYN1	rs3829251	0.28	0.59	G/A^a^	0.297	1.204 (0.863-1.680)	0.342	1.176 (0.841-1.645)
	rs12785878	0.49	0.13	T^a^/G	1.000	0.993 (0.733-1.345)	0.879	0.977 (0.719-1.326)

A/a major allele/minor allele.

^a^risk allele.

^b^adjusted for sex and BMI.

MAF, minor allele frequency in the study population.

HWE, P values for Hardy–Weinberg Equilibrium test in the study population.

^**△**^Significant P values are indicated by a triangle (^**△**^*P*<0.05).

## Results

### Descriptive analysis

In total, 506 individuals were included in this study. The baseline characteristics of these individuals are shown in Table 1. A total of 105 patients had rickets according to the stated criteria and the other 401 individuals were included as controls.

Among the rachitic children in the epidemiological cohort (n = 105), 49% were male. The mean age was 1.77 ± 1.07 years (mean ± SD), the mean BMI was 18.43 ± 4.21, and the mean serum 25(OH)D level was 11.82 ± 6.95 ng/ml.

The control cohort (n = 401) contained significantly more male subjects (63%, *P* = 0.011) and had a similar mean BMI (18.14 ± 3.37, *P*  > 0.05) compared with the rachitic cohort (18.43 ± 4.21). The mean age in control subjects was significantly higher than that of the rachitic subjects (*P* = 4.521 × 10^-38^). However, we had already excluded patients with a history of rickets. Similar to previous reports, serum 25(OH)D levels were much higher in the control cohort than in the rachitic cohort (*P* = 1.335 × 10^-35^) [22]. Biochemical data showed that serum calcium (*P* = 6.355 × 10^-8^) and alkaline phosphatase status (*P* = 0.001) were higher in controls than in rachitic subjects.

### Allelic distribution

We selected and genotyped 12 SNPs significantly associated with 25(OH)D and/or rickets in a recent genome-wide association study, and these SNPs were genotyped in 105 rachitic subjects and 401 control Han individuals from northeastern China. All 12 variants genotyped in the two groups were in Hardy–Weinberg equilibrium. The results are shown in Table 2.

In our case–control cohort, six alleles of 12 SNPs conferred a significantly increased risk of rickets. These included rs4588 C (*P* = 0.003, odds ratio [OR]: 0.583, 95% confidence interval [CI]: 0.412–0.836), rs222020 C (*P* = 0.009, OR: 1.526, 95% CI: 1.117–2.0985), rs2282679 A (*P* = 0.010, OR: 0.636, 95% CI: 0.449–0.900), and rs2298849 C (*P* = 0.001, OR: 1.709, 95% CI: 1.250–2.338) in *GC,* and rs10741657 G (*P* = 0.019, OR: 1.467, 95% CI: 1.070–2.011) and rs2060793 G (*P* = 0.023, OR: 0.689, 95% CI: 0.502–0.944) in *CYP2R1*. The associations of these SNPs with rickets remained significant after adjustment for sex and BMI. The closest association was found in the *GC* variant rs2298849 (adjusted *P* = 0.001). Of the six SNPs associated with rickets in our study, rs4588, rs2282679, and rs2298849 showed the same trend of association as in previous reports of their association with rickets in Caucasian ethnic groups [20].

### Association of different genotypes and rickets

We further analyzed the effect of genotypes under three different genetic models. After adjusting for the confounding variables of sex and BMI, multiple linear regression analysis showed a significant association of all variants with rickets (Table 3).

**Table 3:** Association of different genotypes with rickets in cases and controls

**SNP**	**Genotype**	*p*value^*^			*p*value^**^		
		**Additive**	**Dominant**	**Recessive**	**Additive**	**Dominant**	**Recessive**
rs4588	AA	**4.733 × 10**^**-3△**^	**3.850 × 10**^**-3△**^	0.553	**0.006**^**△**^	**0.005**^**△**^	6.636
	AC						
	CC	0.142			1.704		
rs7041	TT		0.523	0.026		6.276	0.312
	TG	0.965			11.580		
	GG	**0.029**^**△**^			0.348		
rs222020	TT		0.012	0.141		0.144	1.692
	TC	**0.028**^**△**^			0.336		
	CC	**0.028**^**△**^			0.36		
rs2282679	CC	**0.003**^**△**^	0.002	0.576	**0.036**^**△**^	**0.024**^**△**^	6.912
	CA						
	AA	0.180			2.160		
rs2298849	TT	**0.001**^**△**^	**4.193 × 10**^**-3△**^	0.132		**0.005**^**△**^	1.584
	TC				0.012		
	CC	0.010			0.12		
rs10741657	AA		0.227	**0.011**^**△**^		2.724	0.132
	AG	0.724			8.688		
	GG	**0.049**^**△**^			0.588		
rs10766197	AA		0.052	0.223		0.624	2.676
	AG	**0.011**^**△**^			0.132		
	GG	0.794			9.528		
rs12794714	GG		0.154	0.120		1.848	1.440
	GA	0.295			3.540		
	AA	0.064			0.768		
rs1562902	CC		0.449	0.083		5.388	0.996
	CT	0.875			9.420		
	TT	0.163			1.956		
rs2060793	AA	**0.028**^**△**^	**0.014**^**△**^	0.244		0.168	2.928
	AG				0.336		
	GG	0.056			0.672		
rs3829251	GG		0.417	0.465		5.004	5.580
	GA	0.532			6.384		
	AA	0.394			4.728		
rs12785878	GG		0.431	0.576		5.172	6.912
	GT	0.330			3.960		
	TT	0.882			10.584		

*p* value*, p values were adjusted for sex and BMI.

*p* value**, adjusted p values were corrected using the Bonferroni^,^ method.

^**△**^Significant P values are indicated by a triangle (^**△**^*P*<0.05).

As shown in Table 3, five SNPs in the *GC* gene were significantly associated with rickets under three genetic models. However, after applying Bonferroni’s correction for multiple testing, only rs4588, rs2282679, and rs2298849 remained significantly associated with rickets under the dominant (*P* = 0.003 for rs4588, *P* = 0.024 for rs2282679, and *P* = 0.005 for rs2298849) and additive models (*P* = 0.006 for rs4588, *P* = 0.024 for rs2282679, and *P* = 0.005 for rs2298849).

For *CYP2R1*, individuals with rs2060793 of the G allele were found to confer a significantly increased risk for rickets under the additive and dominant models (*P* = 0.028 and *P* = 0.014, respectively). Variants in rs10741657 and rs10766197 showed weak associations with rickets under the additive model (*P* = 0.049 and *P* = 0.011, respectively). However, after correction for multiple testing, none of the investigated SNP associations remained statistically significant.

### Association of haplotypes with rickets

The frequency distribution of the haplotypes and associations with rickets are shown in Table 4. The CAT haplotype of the *GC* gene was significantly associated with rickets (*P* = 0.005). For *CYP2R1*, the GAA haplotype frequency was significantly higher in the case cohort than in the control cohort (*P* = 0.026). No significant difference was observed in the frequency of the *DHCR7* haplotype in cases and controls.

**Table 4:** Frequency distribution of haplotypes and their association with rickets in cases and controls

**Gene**	**Haplotype**^**a**^	**Case (%)**	**Control (%)**	**P value**
GC	ACT	0.469	0.410	0.1205
	CAT	0.229	0.329	**0.005**^**△**^
	ACG	0.288	0.249	0.2452
CYP2R1	GAA	0.352	0.437	**0.0261**^**△**^
	AGG	0.409	0.340	0.06
	GGG	0.238	0.213	0.4281
DHCR7/NADSYN1	TG	0.508	0.503	0.9066
	GA	0.298	0.258	0.2352
	GG	0.187	0.230	0.1867

^a^Loci of tag SNPs are written 3′ to 5′ and include the following SNPs: rs4588, rs7041 and rs2282679 in GC, rs12704714, rs10741657 and 2060793 in CYP2R1 and rs3829251 and rs12785878 in DHCR7/NADSYN1.

^**△**^Significant P values are indicated by a triangle (^**△**^*P*<0.05).

## Discussion

In this study, the association of *GC*, *CYP2R1,* and *DHCR7/NADSYN1* polymorphisms with vitamin D deficient rickets in Chinese subjects was reported for the first time. In this population-based sample, we analyzed the association of 12 loci of three vitamin D-related genes with rickets in Han children from Northeast China. These results suggest that *GC* and *CYP2R1* variants are important contributors to susceptibility to rickets in Chinese children.

GC, also known as vitamin D-binding protein (DBP), and it is a member of the albumin family [23], encodes DBP synthesized in the liver and transports vitamin D and its metabolites. Recently, studies investigated the association between *GC* polymorphisms and 25(OH)D status [24-28]. Because certain mutations in the *GC* gene are known to cause defects in *GC* function, we hypothesized that *GC* polymorphisms can predict susceptibility to developing rickets. In the present study, the strongest association for developing rickets was found to be with a variant at rs2298849. Further analysis showed variant genotypes of SNPs in the *GC* gene. Five polymorphisms were significantly associated with rickets under three genetic models after adjusting for sex and BMI. Even after Bonferroni’s correction, rs4588, rs2282679, and rs2298849 remained significant. Moreover, haplotype analysis suggested that the CAT haplotype of the *GC* gene was significantly associated with a higher risk of rickets. These results indicate that the *GC* gene plays an important role in the pathogenesis of rickets. The possible mechanism may be via impaired vitamin D transportation, although other possibilities cannot be ruled out.

Studies have shown that rs4588 and rs7041 generate functionally different proteins. Such differences affect circulating 25(OH)D concentrations. The rare allele of rs7041 codes for the aspartic acid residue at amino acid position 416 of DBP. The rs4588 allele codes for a lysine residue at position 420, which allows for differentiation of three major DBP phenotypes. Being rare homozygote for both rs7041 and rs4588 characterize the glycosylation pattern of the protein phenotype Gc2-2. This phenotype has been shown to be associated with low mean serum DBP protein concentrations, as well as with low mean serum 25(OH)D concentrations in post-menopausal women. These results suggest that the rare alleles rs7041 and rs4588 are associated with lower 25(OH)D concentrations, at least in part because of the lowering effect on DBP concentrations. Whether the variation in DBP concentrations stems from production of different proteins or degradation rates associated with different DBP genotypes and phenotypes is unclear. Further study is required to explore the potential regulatory mechanisms [25,26].

CYP2R1 is a member of the CYP2 family encoding cytochrome P450 proteins. *CYP2R1* is an important vitamin D 25-hydroxylase that hydroxylates vitamin D at the 25-C position for 25(OH)D synthesis in the liver [29]. Previous clinical studies on *CYP2R1* are limited to only one case report of a Nigerian man with a history of rickets with a point mutation in *CYP2R1 *[30]. In a recent study by Wjst et al. [31], the *CYP2R1* locus was found to be associated with circulating 25-hydroxyvitamin D concentrations, and rs10766197 was significantly associated with 25(OH)D status in 872 participants of the German Asthma Family Study. In addition, Ramos-Lopez et al. [32] found that rs10741657 in *CYP2R1* was associated with the serum status of 25(OH)D in 609 participants from 203 families with type 1 diabetes. Our study found significant evidence that the G allele in rs2060793 and rs10741657 conferred a high risk under genetic models. In our study, haplotype-based associations showed that the significance of associations between haplotypes and rickets is comparable in magnitude with that observed with individual SNPs. The major risk haplotype GAA confers a higher risk than that attributed to other haplotypes in Chinese children. These findings suggest that genetic variants of the *CYP2R1* gene may play an important role in the development of rickets.

The third gene examined, *DHCR7/NADSYN1*, encodes the enzyme 7-dehydrocholesterol (7-DHC) reductase, which transforms 7-DHC to cholesterol. This process leads to removal of that substrate from the synthetic pathway of vitamin D3, a precursor of 25(OH)D. Cooper et al. showed that the rs12785878 T allele was significantly associated with lower levels of 25(OH)D in type 1 diabetic patients [15]. In our study, we found that two variant genotypes of *DHCR7/NADSYN1* (rs3829251 and rs12785878) were associated with serum 25(OH)D levels. However, our study found little evidence that *DHCR7/NADSYN1* variants were associated with a genetic risk of rickets. Genetic heterogeneity among different continental populations due to different geographical locations and genetic histories may explain the inconsistency between these studies. Additionally, differences in the level of environmental risk factors in different populations may alter the effect of susceptibility to loci on the levels of 25(OH)D and the risk of rickets.

There are a few limitations to this study. One limitation was the small size of samples and lack of a replication group. Our findings need to be confirmed in larger samples of Han Chinese children from Northeast China. Considering that the prevalence of rickets is 20%, there is a frequency of 26% to 49% for risk alleles, and an additive genetic model is used, we had 66% to 74% power to detect an OR of 1.50 at the 0.05 level. Another limitation was the lack of a comprehensive genetic analysis. Moreover, we did not identify any clinical differences between patients with or without risk-associated haplotypes because our study focused on young children. Further analyses on patients using a larger sample and people from different ethnicities may be necessary to clarify the mechanism involved.

## Conclusions

In conclusion, our study shows that there are strong effects of *GC* and *CYP2R1* loci on rickets in Han children from northeastern China.

## Competing interests

The authors declare that they have no competing interests.

## Authors’ contributions

YLZ, SFY, and LHR performed variant analysis and interpretation, and drafted the manuscript. YL performed clinical evaluation and assisted in drafting of the manuscript. All authors read and approved the final manuscript.

## Pre-publication history

The pre-publication history for this paper can be accessed here:

http://www.biomedcentral.com/1471-2350/14/101/prepub

## References

[B1] Al-Atawi MS Al-Alwan IA Al-Mutair AN Tamim HM Al-Jurayyan NA Epidemiology of nutritional rickets in children Saudi J Kidney Dis Transpl 2009 20 2 260 265 19237815

[B2] Pettifor JM Nutritional rickets in developing countries Forum Nutr 2003 56 176 178 15806850

[B3] Strand MA Perry J Jin M Tracer DP Fischer PR Zhang P Xi W Li S Diagnosis of rickets and reassessment of prevalence among rural children in northern China Pediatr Int 2007 49 2 202 209 10.1111/j.1442-200X.2007.02343.x 17445039

[B4] Baroncelli GI Bereket A El Kholy M Audi L Cesur Y Ozkan B Rashad M Fernandez-Cancio M Weisman Y Saggese G Rickets in the Middle East: role of environment and genetic predisposition J Clin Endocrinol Metab 2008 93 5 1743 1750 10.1210/jc.2007-1413 18285415

[B5] Bora G Ozkan B Dayangac-Erden D Erdem-Yurter H Coskun T Vitamin D receptor gene polymorphisms in Turkish children with vitamin D deficient rickets Turk J Pediatr 2008 50 1 30 33 18365588

[B6] Fischer PR Thacher TD Pettifor JM Jorde LB Eccleshall TR Feldman D Vitamin D receptor polymorphisms and nutritional rickets in Nigerian children J Bone Miner Res 2000 15 11 2206 2210 10.1359/jbmr.2000.15.11.2206 11092401

[B7] Kaneko A Urnaa V Nakamura K Kizuki M Seino K Inose T Takano T Vitamin D receptor polymorphism among rickets children in Mongolia J Epidemiol 2007 17 1 25 29 10.2188/jea.17.25 17202743 PMC7058450

[B8] Gong YG Li YN Zhang WH Liu LJ Kang XG Correlation between vitamin D receptor genetic polymorphism and 25-hydroxyvitamin D3 in vitamin D deficiency rickets Zhongguo Dang Dai Er Ke Za Zhi 2010 12 7 544 546 20637153

[B9] Lu HJ Li HL Hao P Li JM Zhou LF Association of the vitamin D receptor gene start codon polymorphism with vitamin D deficiency rickets Zhonghua Er Ke Za Zhi 2003 41 7 493 496 14746673

[B10] Lu JJ Li YN Jin Y Li L Association of the vitamin D receptor gene start codon polymorphism with delayed rickets Zhonghua Er Ke Za Zhi 2007 45 1 46 49 17349151

[B11] Wu SH Yan CH Shen XM Vitamin D receptor gene polymorphisms and vitamin D deficiency rickets Zhongguo Dang Dai Er Ke Za Zhi 2006 8 1 83 inside back cover 16522251

[B12] Wu SH Yu XD Yan CH Shen LX Yu XG Zhang YP Zhang JS Jin XM Shen XM Association between vitamin D receptor gene polymorphism and vitamin D deficiency rickets Zhongguo Dang Dai Er Ke Za Zhi 2006 8 2 121 124 16613705

[B13] Xi WP Yang JP Li LQ Zhu QY Zhou XH Association of vitamin D receptor gene Apa I polymorphism with vitamin D deficiency rickets Zhonghua Er Ke Za Zhi 2005 43 7 514 516 16083553

[B14] Cheng JB Motola DL Mangelsdorf DJ Russell DW De-orphanization of cytochrome P450 2R1: a microsomal vitamin D 25-hydroxilase J Biol Chem 2003 278 39 38084 38093 10.1074/jbc.M307028200 12867411 PMC4450819

[B15] Cooper JD Smyth DJ Walker NM Stevens H Burren OS Wallace C Greissl C Ramos-Lopez E Hypponen E Dunger DB Inherited variation in vitamin D genes is associated with predisposition to autoimmune disease type 1 diabetes Diabetes 2011 60 5 1624 1631 10.2337/db10-1656 21441443 PMC3292339

[B16] White P Cooke N The multifunctional properties and characteristics of vitamin D-binding protein Trends Endocrinol Metab 2000 11 8 320 327 10.1016/S1043-2760(00)00317-9 10996527

[B17] Kurylowicz A Ramos-Lopez E Bednarczuk T Badenhoop K Vitamin D-binding protein (DBP) gene polymorphism is associated with Graves’ disease and the vitamin D status in a Polish population study Exp Clin Endocrinol Diabetes 2006 114 6 329 335 10.1055/s-2006-924256 16868893

[B18] Ramos-Lopez E Bruck P Jansen T Herwig J Badenhoop K CYP2R1 (Vitamin D 25-hydroxylase) gene is associated with susceptibility to type 1 diabetes and vitamin D levels in Germans Diabetes Metab Res Rev 2007 23 8 631 636 10.1002/dmrr.719 17607662

[B19] Wang TJ Zhang F Richards JB Kestenbaum B van Meurs JB Berry D Kiel DP Streeten EA Ohlsson C Koller DL Common genetic determinants of vitamin D insufficiency: a genome-wide association study Lancet 2010 376 9736 180 188 10.1016/S0140-6736(10)60588-0 20541252 PMC3086761

[B20] Bu FX Armas L Lappe J Zhou Y Gao G Wang HW Recker R Zhao LJ Comprehensive association analysis of nine candidate genes with serum 25-hydroxy vitamin D levels among healthy Caucasian subjects Hum Genet 2010 128 5 549 556 10.1007/s00439-010-0881-9 20809279

[B21] Purcell SCS Sham PC Genetic power calculator: design of linkage and association genetic mapping studies of complex traits Bioinformatics 2003 19 149 150 10.1093/bioinformatics/19.1.149 12499305

[B22] Baroncelli GI Bereket A El Kholy M Audì L Cesur Y Ozkan B Rashad M Fernández-Cancio M Weisman Y Saggese G Hochberg Z Rickets in the Middle East: role of environment and genetic predisposition J Clin Endocrinol Metab 2008 93 5 1743 1750 10.1210/jc.2007-1413 18285415

[B23] Speeckaert M Huang G Delanghe JR Taes YE Biological and clinical aspects of the vitamin D binding protein (Gc-globulin) and its polymorphism Clin Chim Acta 2006 372 1–2 33 42 16697362 10.1016/j.cca.2006.03.011

[B24] Ahn J Albanes D Berndt SI Peters U Chatterjee N Freedman ND Abnet CC Huang WY Kibel AS Crawford ED Vitamin D-related genes, serum vitamin D concentrations and prostate cancer risk Carcinogenesis 2009 30 5 769 776 10.1093/carcin/bgp055 19255064 PMC2675652

[B25] Abbas S Linseisen J Slanger T Kropp S Mutschelknauss EJ Flesch-Janys D Chang-Claude J The Gc2 allele of the vitamin D binding protein is associated with a decreased postmenopausal breast cancer risk, independent of the vitamin D status Cancer Epidemiol Biomarkers Prev 2008 17 6 1339 1343 10.1158/1055-9965.EPI-08-0162 18559548

[B26] Sinotte M Diorio C Berube S Pollak M Brisson J Genetic polymorphisms of the vitamin D binding protein and plasma concentrations of 25-hydroxyvitamin D in premenopausal women Am J Clin Nutr 2009 89 2 634 640 10.3945/ajcn.2008.26445 19116321

[B27] Fu L Yun F Oczak M Wong BY Vieth R Cole DE Common genetic variants of the vitamin D binding protein (DBP) predict differences in response of serum 25-hydroxyvitamin D [25(OH)D] to vitamin D supplementation Clin Biochem 2009 42 10–11 1174 1177 19302999 10.1016/j.clinbiochem.2009.03.008

[B28] Fang Y van Meurs JB Arp P van Leeuwen JP Hofman A Pols HA Uitterlinden AG Vitamin D binding protein genotype and osteoporosis Calcif Tissue Int 2009 85 2 85 93 10.1007/s00223-009-9251-9 19488670 PMC2729412

[B29] Shinkyo R Sakaki T Kamakura M Ohta M Inouye K Metabolism of vitamin D by human microsomal CYP2R1 Biochem Biophys Res Commun 2004 324 1 451 457 10.1016/j.bbrc.2004.09.073 15465040

[B30] Cheng JB Levine MA Bell NH Mangelsdorf DJ Russell DW Genetic evidence that the human CYP2R1 enzyme is a key vitamin D 25-hydroxylase Proc Natl Acad Sci U S A 2004 101 20 7711 7715 10.1073/pnas.0402490101 15128933 PMC419671

[B31] Wjst M Altmuller J Faus-Kessler T Braig C Bahnweg M Andre E Asthma families show transmission disequilibrium of gene variants in the vitamin D metabolism and signalling pathway Respir Res 2006 7 60 10.1186/1465-9921-7-60 16600026 PMC1508148

[B32] Ramos-Lopez E Bruck P Jansen T Herwig J Badenhoop K CYP2R1 (Vitamin D 25-hydroxylase) gene is associated with susceptibility to type 1 diabetes and vitamin D levels in Germans Diabetes Metab Res Rev 2007 23 8 631 636 10.1002/dmrr.719 17607662

